# Clinical and radiological factors associated with unfavorable outcome after intravenous thrombolysis in patients with mild ischemic stroke

**DOI:** 10.1186/s12883-018-1033-4

**Published:** 2018-03-15

**Authors:** Dae-Hyun Kim, Deok-Soo Lee, Hyun-Wook Nah, Jae-Kwan Cha

**Affiliations:** 1 Busan-Ulsan Regional Cardiocerebrovascular Center, Dong-A University Hospital, Busan, Republic of Korea; 2 Department of Neurology, College of Medicine, Dong-A University, 1, 3-ga Dongdaesin-dong, Seo-gu, Busan, 602-715 Republic of Korea

**Keywords:** Mild ischemic stroke, Recombinant tissue plasminogen activator, Diffusion-weighted imaging

## Abstract

**Background:**

A significant proportion of patients with mild ischemic stroke become disabled despite receiving intravenous thrombolytic therapy. The purpose of this study was to assess the clinical and radiological factors associated with unfavorable outcomes in patients with minor ischemic stroke that received intravenous recombinant tissue plasminogen activator (rt-PA) therapy.

**Methods:**

We identified anterior circulation stroke patients with initial National Institutes of Health Stroke Scale (NIHSS) scores ≤5 who received intravenous thrombolysis within 4.5 h of stroke onset and had pretreatment magnetic resonance (MR)/MR angiography using our prospective stroke database. We analyzed baseline characteristics, infarction patterns on diffusion-weighted imaging (DWI), and steno-occlusive lesions on MR angiography. Unfavorable outcome was defined as a modified Rankin Scale (mRS) score ≥ 2 at 90 days. Logistic regression was used to determine independent predictors of unfavorable outcomes.

**Results:**

Among 121 patients (85 men; mean age, 63.4 ± 11.3 years) included in this study, 46 (38%) had unfavorable outcomes at 90 days and DWI lesion patterns showing infarction in the deep middle cerebral artery (MCA) territory involving the perforating artery area was observed in 47 (38.8%) patients. On multivariable analysis, unfavorable outcomes at 90 days were associated with diabetes [odds ratio (OR), 3.41; 95% confidence interval (CI), 1.06–10.9; *P* = 0.039), NIHSS score on admission (OR, 2.11; 95% CI, 1.35–3.30; *P* = 0.001), and infarction in the deep MCA territory on DWI (OR, 4.19; 95% CI, 1.63–10.8; *P* = 0.003). Lesions in the deep MCA territory was independently associated with early neurological deterioration (*P* = 0.032). The patients without deep MCA territory infarction had a higher prevalence of cardiac embolism (*P* = 0.009).

**Conclusions:**

Higher NIHSS scores, diabetes, and deep MCA territory infarction may be useful for predicting unfavorable outcomes in patients with minor stroke treated with intravenous rt-PA therapy.

**Electronic supplementary material:**

The online version of this article (10.1186/s12883-018-1033-4) contains supplementary material, which is available to authorized users.

## Background

More than half of all ischemic stroke patients exhibit a clinical syndrome with mild neurological deficit [1, 2]. Intravenous thrombolysis with recombinant tissue plasminogen activator (rt-PA) within 4.5 h has been proven to be an effective treatment for acute ischemic stroke, but its benefits and risks in mild strokes are still unclear [3]. When patients with a mild stroke received intravenous thrombolytic therapy, approximately 30% of them had an unfavorable outcome [3–5]. Thus, it is of great importance to identify the patients at risk of unfavorable outcomes in the acute phase to prevent neurological deterioration and to predict outcomes in minor ischemic strokes treated with intravenous thrombolysis.

Magnetic resonance imaging (MRI) with diffusion-weighted imaging (DWI) is superior to computed tomography (CT) for early detection of hyperacute and small ischemic lesions. Pretreatment DWI lesion patterns may be helpful in predicting clinical outcome in patients with acute ischemic stroke [6, 7]. Steno-occlusive lesions of the cerebral artery are associated with unfavorable outcomes in patients with minor strokes [8, 9]. However, it is unclear whether the pretreatment lesions pattern on DWI and the presence of steno-occlusive lesions in relevant arteries can predict outcome following rt-PA thrombolysis in patients with minor stroke.

The aim of our study was to elucidate the clinical and pretreatment radiological factors associated with an unfavorable outcome after intravenous thrombolysis in patients with minor stroke.

## Methods

We identified 682 consecutive patients in our prospectively collected institutional stroke database treated with intravenous rt-PA therapy within 4.5 h after symptom onset, independent of performance of emergent endovascular treatment between January 2010 and December 2016. During this period, MRI was systematically adopted at our center as the first-line imaging modality [DWI, fluid-attenuated inversion recovery, gradient echo sequences, and time of flight magnetic resonance angiography (MRA)] in the absence of contraindications to MRI. Eligible patients were treated with intravenous rt-PA according to clinical guidelines. No upper or lower National Institutes of Health Stroke Scale (NIHSS) threshold was applied. Follow up MRI or CT were performed 24 h after intravenous rt-PA thrombolysis in all patients.

Patients were included in our study if they had an anterior circulation stroke with an initial NIHSS score of 1 to 5 and pretreatment MRI including DWI and MRA. We excluded patients who; (1) had a pre-stroke modified Rankin scale core (mRS) > 1, (2) received subsequent endovascular treatment combined with intravenous thrombolysis, (3) had no definitive evidence of focal hyperintensities in clinically relevant areas on initial or follow up DWI, or (4) had no follow-up data.

Demographic, clinical and laboratory data were collected from the prospectively collected stroke registry. Baseline data included initial NIHSS scores and onset-to-rt-PA treatment times. Mild stroke was defined as a baseline NIHSS score 1–5 [5]. The etiologic stroke subtype was defined according to the Trial of Org 10,172 in Acute Stroke Treatment (TOAST) criteria [10].

This study was approved by the Institutional Review Board of Dong-A University Hospital.

### Imaging analysis

**Fig. 1:**
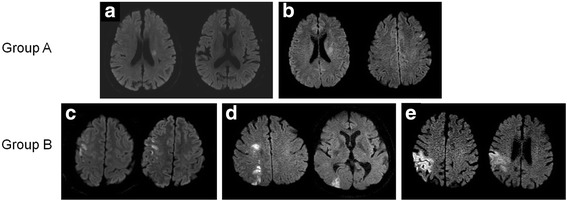
Examples of the five different diffusion-weighted imaging patterns analyzed and the classification into two groups. Lesion patterns were classified as perforating artery infarcts (**a**), perforating artery infarcts with additional infarcts outside the perforating artery territory (**b**), pial infarcts (**c**), border-zone infarcts (**d**) and territorial infarcts (**e**)

Two investigators (D.-H. K and D.-S. L) who were blinded to the patients’ clinical characteristics reviewed the pretreatment DWI and MRA images and follow up DWI at 24 h after intravenous thrombolysis. Discrepancies were resolved by consensus. DWI lesion pattern were classified as perforating artery infarcts (PAI), pial infarcts, border-zone infarcts, territorial infarcts and PAI plus additional infarcts outside the perforating artery territory, by modification of previous studies (Fig. 1) [11].

These findings were also divided into two different groups according to the presence of infarcts in the territory of the perforating arteries involving the internal capsule, corona radiata, and basal ganglia. Deep middle cerebral artery (MCA) infarction with or without infarctions outside the perforating artery territory were classified as Group A while the other 3 lesion patterns were classified as Group B.

Stenosis or occlusion of the arteries related to ischemia were determined via analysis of the initial MRA, which included the entire internal carotid artery, MCA (M1 proximal, M1 distal, and M2 segment), and anterior cerebral artery (ACA; A1 segment). Arterial occlusion was defined as a complete loss of distal flow signal. Moderate to severe arterial stenosis was defined as a 50% narrowing of the lumen and focal signal loss in the presence of a distal flow signal.

### Clinical outcomes

Early neurological deterioration (END) was defined as an increase of 2 or more points in the NIHSS scores between hospital days 0 and 5. Outcome was assessed using the mRS score at 3 months from stroke onset and was dichotomized into favorable (mRS 0–1) and unfavorable (mRS 2–6). Symptomatic intracerebral hemorrhage was defined as blood at any site in the brain associated with clinical deterioration resulting in an NIHSS score increase of ≥4 points [12].

### Statistical analysis

The results are expressed as mean (standard deviation) or median (range), as appropriate, throughout the text and tables. The differences in patients’ characteristics and their relation to 90-day outcomes (mRS ≤ 1 vs. mRS ≥ 2) were analyzed. For dichotomous outcomes, the chi-squared or Fisher’s exact test are reported where appropriate. For outcomes with continuous data, comparisons were performed using Student’s t test or the Mann-Whitney U test, depending on the normality of data distribution. Multivariate logistic regression analysis was performed to determine factors that could be considered to be independent predictors of unfavorable outcome after intravenous rt-PA thrombolysis. Variables showing a value of *P* < 0.2 in univariate analysis were included in the multivariate model. A probability value < 0.05 was considered statistically significant.

## Results

**Fig. 2:**
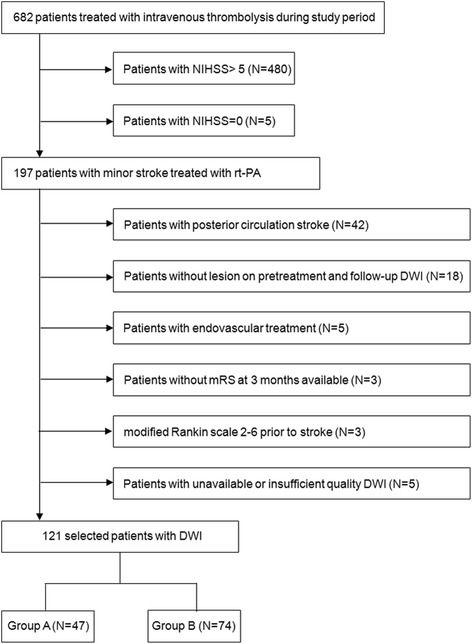
Schematic description of the patient selection process

A total of 202 patients with baseline NIHSS ≤5 were treated with intravenous thrombolysis. We excluded 42 patients with posterior circulation strokes, 18 patients without hyperintensity lesions on pretreatment and follow-up DWI, 5 patients who received endovascular treatment, 5 patients with an initial NIHSS of 0, 5 patients with unavailable or insufficient quality DWI, 3 patients with mRS 2–6 prior to the stroke, and 3 patients without an mRS assessment at 3 months Finally, 121 patients (85 men and 36 women; mean age, 63.4 ± 11.3 years) were included in the study (Fig. 2). The median baseline NIHSS score was 4 (interquartile range, 3–5). Among 121 patients, 46 (38%) had unfavorable outcomes (mRS score 2–6) at 90 days. Symptomatic intracerebral hemorrhages were rare (1.7%).

Table 1 presents the general characteristics and radiologic features of patients with and without unfavorable outcomes. In univariate analysis, diabetes (*P* = 0.007), non-cardiac embolism (*P* = 0.04), higher NIHSS scores (*P* = 0.003), and deep MCA territory infarction (*P* < 0.001) were more frequently observed in patients with an unfavorable outcome. The onset-to-rt-PA time was 141.6 ± 58 and 145.6 ± 67 min in the favorable and unfavorable groups, respectively, and did not differ significantly (*P* = 0.728). Visible arterial occlusion before rtPA was observed in 33% of patients. They were comprised of ICA occlusion (*N* = 8), M1 proximal occlusion (*N* = 10), M1 distal occlusion (*N* = 11), M2 occlusion (*N* = 9), and ACA occlusion (*N* = 2) on MRA. There was no difference in favorable outcome following intravenous thrombolysis between mild stroke patients with and without large artery occlusion.

**Table 1:** Characteristics of the study population according to outcomes at 3 months

	All patients (*n* = 121)	mRS = 0–1 (*n* = 75)	mRS = 2–6 (*n* = 46)	*P*-value
Age (mean ± SD)	63.4 ± 11.3	63.2 ± 10.5	63.8 ± 12.6	0.769
Sex, *n*(%)				0.490
Men	85(70.2)	51(68)	34(73.9)	
Female	36(29.8)	24(32)	12(26.1)	
Risk factor, n(%)				
Hypertension	61(50.8)	36(48.6)	25(54.3)	0.544
Diabetes	33(27.3)	14(18.7)	19(41.3)	0.007
Hyperlipidemia	17(14)	7(9.3)	10(21.7)	0.057
Atrial fibrillation	22(18.2)	16(21.3)	6(13)	0.251
Current smoking	31(25.6)	20(26.7)	11(23.9)	0.736
Previous coronary artery disease	14(11.6)	10(13.3)	4(8.7)	0.564
Previous stroke or TIA	11(9.1)	6(8.0)	5(10.9)	0.746
NIHSS on admission, (med, IQR)	4[3–5]	4[2–4]	4[3–5]	0.003
(mean ± SD)	3.6 ± 1.2	3.4 ± 1.2	4.0 ± 1.1	0.004
Onset to rt-PA time, (mean± SD)	143.1 ± 61.4	141.6 ± 57.9	145.6 ± 67.2	0.728
Blood glucose on admission, mg/dL	143.4 ± 52.6	134.7 ± 44.9	157.6 ± 61.0	0.019
Initial systolic blood pressure, mmHg	145.6 ± 26.3	145.4 ± 27.9	145.8 ± 23.5	0.924
Etiologic stroke subtypes, *n*(%)				0.302
Small vessel disease	25(20.7)	13(17.3)	12(26.1)	0.248
Large artery disease	36(29.8)	22(29.3)	14(30.4)	0.298
Cardiac embolism	34(28.1)	26(34.7)	8(17.4)	0.040
Undetermined etiology	26(21.5)	14(18.7)	12(26.1)	0.335
Lesions on pretreatment DWI, *n*(%)				0.317
Acute infarcts (+)	110(91.7)	67(89.3)	43(95.6)	
No lesions	10(8.3)	8(10.7)	2(4.4)	
Lesion patterns on DWI, *n*(%)				
Perforating artery infarcts	27(22.3)	12(16)	15(32.6)	0.033
Pial infarcts	51(42.1)	40(53.3)	11(23.9)	0.001
Borderzone infarcts	6(5)	5(6.7)	1(2.2)	0.406
Territorial infarcts	17(14)	10(13.3)	7(15.2)	0.772
Perforating artery infarcts plus	20(16.5)	8(10.7)	12(26.1)	0.027
Deep MCA infarcts (PAI + PAI plus)	47(38.8)	20(26.7)	27(58.7)	< 0.001
Angiographic findings of relevant artery, *n*(%)				0.383
No stenosis or occlusion	62(51.2)	42(56)	20(43.5)	
Stenosis (> 50%)	19(15.7)	10(13.3)	9(19.6)	
Occlusion	40(33.1)	23(30.7)	17(37)	
Steno-occlusive disease, *n*(%)	59(48.8)	33(44)	26(56.5)	0.181
END, *n*(%)	14(11.6)	3(4)	11(23.9)	0.002
Symptomatic ICH, *n*(%)	2(1.7)	1(1.3)	1(2.2)	1.000

*TIA* transient ischemic attack, *DWI* diffusion-weighted imaging, MCA middle cerebral artery, *PAI* perforating artery infarction, *END* early neurological deterioration, ICH intracranial hemorrhage

After univariate analysis, hyperlipidemia (*P* = 0.058), age, sex, previous stroke, and steno-occlusive disease (*P* = 0.181) were also included in the multivariate logistic regression. Multivariate logistic regression analysis showed that diabetes [odds ratio (OR), 3.41; 95% confidence interval (CI), 1.06–10.9; *P* = 0.039], higher NIHSS scores on admission (OR, 2.11; 95% CI, 1.35–3.30; *P* = 0.001), and infarction in the deep MCA territory (OR, 4.19; 95% CI, 1.63–10.8; *P* = 0.003) were independently associated with unfavorable outcome (Table 2).

**Table 2:** Multivariable analysis for unfavorable outcome at 90 days

	Unfavorable outcome (mRS score 2–6)		
	Adjusted OR	95% CI	*P*-value
Age - per 1 year	1.01	0.96–1.05	0.914
Men	1.95	0.69–5.48	0.205
Diabetes	3.41	1.06–10.9	0.039
Hyperlipidemia	2.92	0.81–10.5	0.102
Previous stroke or TIA	1.29	0.29–5.65	0.755
NIHSS on admission	2.11	1.35–3.30	0.001
Initial serum glucose	1.01	0.99–1.01	0.306
Cardiac embolism	0.48	0.16–1.46	0.195
Presence of deep MCA infarction	4.19	1.63–10.8	0.003
Steno-occlusive disease	1.6	0.63–3.72	0.351

Adjusted by age, sex and previous stroke

**Fig. 3:**
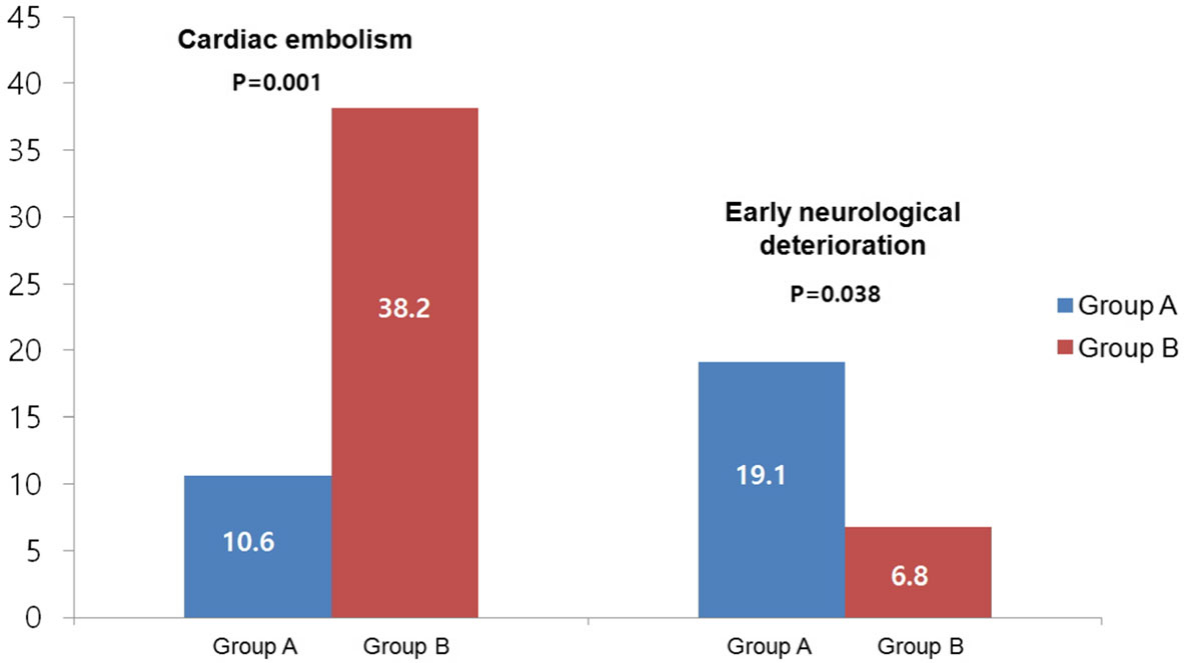
Comparison of the general characteristics of group (**a**) and (**b**)

A DWI lesion pattern with infarction in the deep MCA territory (Group A) was observed in 47 (38.8%) patients. Group A was independently associated with END (*P* = 0.032) and moderate to severe (> 50%) stenosis in the relevant artery (*P* = 0.019). The patients without lesions in the deep MCA territory (Group B) had a higher prevalence of cardiac embolism (*P* = 0.009) (Additional file 1: Table S1, Fig. 3).

## Discussion

This study showed that a large proportion (38%) of patients receiving intravenous thrombolysis for minor strokes will have an unfavorable outcome at 90 days. Symptomatic intracerebral hemorrhages were rare. Baseline NIHSS score, diabetes mellitus, and deep MCA infarction with lesions in the perforating artery territory were independently associated with unfavorable outcomes.

According to recent reports, age [5, 13], diabetes mellitus [5, 8, 9, 14], previous stroke [14], intra-or extracranial vascular occlusive lesions [8, 9] and NIHSS score [5, 15] were associated with unfavorable outcomes in patients with minor strokes. However, these studies explored the factors associated with disability at 90 days in mild stroke patients not treated with thrombolytic therapy.

It remains to be determined whether the presence of steno-occlusive lesions leads to unfavorable outcomes in mild stroke patients following intravenous rt-PA treatment. As opposed to previous reports on non-thrombolytic therapy [8, 9], we did not find an association between large artery occlusion and unfavorable outcome following intravenous rt-PA therapy. This is in line with previous reports that also found no differences in outcome following intravenous thrombolysis between mild stroke patients with and without large artery occlusion [16, 17]. Mild stroke patients tend to have more distal artery occlusion, lower thrombus burden, and higher recanalization rates or have central occlusions with good collaterals [18–20]. This might be the reason why even patients with large artery occlusion do not have different outcome following intravenous rt-PA therapy compared to those without.

Previous studies have shown that acute lesion patterns may predict prognosis or stroke recurrence after ischemic stroke [11, 21] but it remains unclear whether such imaging factors are related to patient outcome after intravenous thrombolysis in mild ischemic stroke. Patients with perforating artery infarcts in the basal ganglia and corona radiata were more likely to have unfavorable outcomes in this study. These finding may be explained by several hypothesis.

First, Strambo et al. [15] reported that among all NIHSS items, only impairment of motor items is significantly associated with poor outcome in mild stroke. Since the deep MCA territory is crossed by the corticospinal tract [22] and is prone to damage by poor collateral flow [23], this area may be susceptible to motor deficits, regardless of rt-PA thrombolysis. In some reports, patients with deep white matter or basal ganglia infarction have suffered from bad outcomes after thrombolysis or endovascular therapy [7, 22, 23]. Thus, even small lesions in this area could be critical to the outcome of mild ischemic stroke.

Second, END is an important factor associated with worse clinical outcome in minor strokes [9]. Several patients with infarcts in the perforator territory have END (29.1%) [13] and this rate was similar to that in patients with small vessel disease that received intravenous rt-PA therapy (33%) [24]. In our study, ENDs were more prevalent in Group A patients and were associated with unfavorable outcomes. These results may have important clinical implications. Previous study has shown that rt-PA use in acute lacunar stroke does not affect END [24]. Thus, special attention should be paid to patients with infarcts in the perforating territory, even when they show mild neurological deficits at the time of presentation and after rt-PA therapy.

Lastly, Group B was more likely to have cardiac embolism. This can be explained by a previous report that demonstrated a mild stroke caused by cardiac embolism could reveal small cortical lesions and lead to a high rate of neurological improvement [15]. However, Hao et al. [25] reported that in mild stroke, cardiac embolism was a predictor of death or disability at 3 months and that patients with small vessel occlusion had better outcomes. That study enrolled mild stroke patients within 30 days after stroke onset. There is a possibility that non-cardiac embolism patients with early worsening or early recurrence were excluded from that study, whereas we included patients treated with rt-PA therapy within 4.5 h. Further studies are needed to confirm whether cardiac embolism can be a protective factor in ischemic stroke with mild symptoms.

A significant strength of our study is that all patients underwent pretreatment MRI. DWI has the advantage of capturing small infarctions and detection of early ischemic changes in patients with acute mild ischemic stroke, unlike CT. Few studies on whether pretreatment DWI lesions pattern and arterial occlusion on MRA can predict outcome in mild stroke are available [21, 26]. To the best of our knowledge, this is the largest study to investigate the DWI findings in patients with mild stroke treated with rt-PA therapy.

The most notable limitation of our study is the uncontrolled, non-randomized design since this was a single hospital-based study. Limitations to using clinical registry data may include the introduction of bias in patient selection including the imbalance of sex ratio in this study. Thus, generalizing the results of this study to other patient populations may be challenging.

## Conclusions

We found that in spite of intravenous rt-PA treatment, approximately 40% of anterior circulation stroke patients with minor symptom had unfavorable outcomes at 3 months. Higher NIHSS scores, diabetes and deep MCA infarction with involvement of the perforating artery territory were associated with unfavorable outcomes after rt-PA therapy in patients with minor stroke. Brain MRI-DWI patterns assessed in the acute phase of mild stroke are useful to predict those patients at high risk of unfavorable outcome following rt-PA therapy.

## Additional file

Additional file 1: **Table S1.** Univariate and multivariable analysis of factors associated with Group A lesion pattern in diffusion-weighted imaging. (DOCX 18 kb)

## Abbreviations

ACA: Anterior cerebral artery
CT: Computed tomography
DWI: Diffusion-weighted imaging
END: Early neurological deterioration
MCA: Middle cerebral artery
MRA: Magnetic resonance angiography
MRI: Magnetic resonance imaging
mRS: modified Rankin scale core
NIHSS: National Institutes of Health Stroke Scale
PAI: Perforating artery infarcts
rt-PA: recombinant tissue plasminogen activator
TOAST: Trial of Org 10,172 in Acute Stroke Treatment
