# Supplementary material for: Clinical and radiological factors associated with unfavorable outcome after intravenous thrombolysis in patients with mild ischemic stroke
Source: BMC Neurol. 2018 Mar 15;18:30. doi: 10.1186/s12883-018-1033-4 (PMC5856376; doi:10.1186/s12883-018-1033-4)
Supplement: Supplementary file 1 — Table S1. Univariate and multivariable analysis of factors associated with Group A lesion pattern in diffusion-weighted imaging. (DOCX 18 kb) [file 12883_2018_1033_MOESM1_ESM.docx]

Table S1. Univariate and multivariable analysis of factors associated with Group A lesion pattern in diffusion-weighted imaging

|  | Group A  (n=47) | Group B  (n=74) | P-value | Multivariable | |
| --- | --- | --- | --- | --- | --- |
|  |  |  |  | OR (95% CI) | P-value |
| Age (mean±SD) | 63.6±12.6 | 63.3±10.4 | 0.878 | - | - |
| Female, n(%) | 16(34) | 20(27) | 0.411 | - | - |
| Risk factor, n(%) |  |  |  | - | - |
| Hypertension | 21(45.7) | 40(54.1) | 0.371 | - | - |
| Diabetes | 15(31.9) | 18(24.3) | 0.361 | - | - |
| Hyperlipidemia | 7(14.9) | 10(13.5) | 0.831 | - | - |
| Atrial fibrillation | 5(10.6) | 17(23) | 0.086 | 7.37(0.69-79.1) | 0.099 |
| Current smoking | 13(27.7) | 18(24.3) | 0.682 | - | - |
| Previous coronary artery disease | 2(4.3) | 12(16.2) | 0.045 | 0.35(0.07-1.92) | 0.227 |
| Previous stroke or TIA | 4(8.5) | 7(9.5) | 0.860 | - | - |
| NIHSS on admission, (med, IQR) | 4[3-5] | 4[3-5] | 0.943 | - | - |
| (mean±SD) | 3.5±1.2 | 3.7±1.2 | 0.964 | - | - |
| Onset to rt-PA time, (med, IQR) | 152.7±62.1 | 147.3±104.6 | 0.747 | - | - |
| Blood glucose on admission, mg/dL | 152.3±54.8 | 137.8±50.7 | 0.138 | - | - |
| Initial systolic blood pressure, mmHg | 150.4±26.9 | 142.5±25.6 | 0.106 | - | - |
| Cardiac embolism, n(%) | 5(10.6) | 29(39.2) | 0.001 | 0.05(0.01-0.49) | 0.009 |
| Angiographic findings of relevant artery, n(%) |  |  | 0.016 |  |  |
| No stenosis or occlusion | 21(44.7) | 41(55.4) |  | Ref | Ref |
| Stenosis (>50%) | 13(27.7) | 6(8.1) |  | 4.77(1.30-17.5) | 0.019 |
| Occlusion | 13(27.7) | 27(36.5) |  | 0.63(0.24-1.67) | 0.354 |
| Steno-occlusive disease, n(%) | 33(44.6) | 26(56.5) | 0.250 | - | - |
| Early neurological deterioration, n(%) | 9(19.1) | 5(6.8) | 0.038 | 4.57(1.14-18.3) | 0.032 |
| Symptomatic ICH, n(%) | 1(2.1) | 1(1.4) | 0.744 | - | - |

TIA; transient ischemic attack, ICH; intracranial hemorrhage
